# YOLOv12 Algorithm-Aided Detection and Classification of Lateral Malleolar Avulsion Fracture and Subfibular Ossicle Based on CT Images: Multicenter Study

**DOI:** 10.2196/79064

**Published:** 2025-10-03

**Authors:** Jiayi Liu, Peng Sun, Yousheng Yuan, Zihan Chen, Ke Tian, Qian Gao, Xiangsheng Li, Liang Xia, Jun Zhang, Nan Xu

**Affiliations:** 1 Department of Radiology, Sir Run Run Hospital, Nanjing Medical University Nanjing China; 2 Department of Radiology, Air Force Medical Center, Air Force Medical University Beijing China; 3 School of Mathematics and Physics, Xi'an Jiaotong-Liverpool University Suzhou China

**Keywords:** deep convolutional neural network, lateral malleolar avulsion fracture, subfibular ossicle, X-ray computed tomography, CT, classification, artificial intelligence, AI

## Abstract

**Background:**

Lateral malleolar avulsion fractures (LMAFs) and subfibular ossicles (SFOs) are distinct entities that both present as small bone fragments near the lateral malleolus in imaging but require different treatment strategies. Clinical and radiological differentiation is challenging, which can impede timely and precise management. Magnetic resonance imaging (MRI) is the diagnostic gold standard for differentiating LMAFs from SFOs, whereas radiological differentiation using computed tomography (CT) alone is challenging in routine practice. Deep convolutional neural networks (DCNNs) have shown promise in musculoskeletal imaging diagnostics, but robust, multicenter evidence in this specific context is lacking.

**Objective:**

This study aims to evaluate several state-of-the-art DCNNs—including the latest You Only Look Once (YOLO) v12 algorithm—for detecting and classifying LMAFs and SFOs in CT images, using MRI-based diagnoses as the gold standard and to compare model performance with radiologists reading CT alone.

**Methods:**

In this retrospective study, 1918 patients (LMAF: n=1253, 65.3%; SFO: n=665, 34.7%) were enrolled from 2 hospitals in China between 2014 and 2024. MRI served as the gold standard and was independently interpreted by 2 senior musculoskeletal radiologists. Only CT images were used for model training, validation, and testing. CT images were manually annotated with bounding boxes. The cohort was randomly split into a training set (n=1092, 56.93%), internal validation set (n=476, 24.82%), and external test set (n=350, 18.25%). Four deep learning models—faster R-CNN, single shot multibox detector (SSD), RetinaNet, and YOLOv12—were trained and evaluated using identical procedures. Model performance was assessed using mean average precision at intersection over union=0.5 (mAP50), area under the receiver operating curve (AUC), accuracy, sensitivity, and specificity. The external test set was also independently interpreted by 2 musculoskeletal radiologists with 7 and 15 years of experience, with results compared with the best-performing model. Saliency maps were generated using Shapley values to enhance interpretability.

**Results:**

Among the evaluated models, YOLOv12 achieved the highest detection and classification performance, with a mAP50 of 92.1% and an AUC of 0.983 on the external test set—significantly outperforming faster R-CNN (mAP50 63.7%; AUC 0.79); SSD (mAP50 63%; AUC 0.63); and RetinaNet (mAP50 67.0%; AUC 0.73)—all *P*<.001. When using CT alone, radiologists performed at a moderate level (accuracy: 75.6% and 69.1%; sensitivity: 75.0% and 65.2%; specificity: 76.0% and 71.1%), whereas YOLOv12 approached MRI-based reference performance (accuracy: 92.0%; sensitivity: 86.7%; specificity: 82.2%). Saliency maps corresponded well with expert-identified regions.

**Conclusions:**

While MRI (read by senior radiologists) is the gold standard for distinguishing LMAFs from SFOs, CT-based differentiation is challenging for radiologists. A CT-only DCNN (YOLOv12) achieved substantially higher performance than radiologists interpreting CT alone and approached the MRI-based reference standard, highlighting its potential to augment CT-based decision-making where MRI is limited or unavailable.

## Introduction

### Background

The ankle is the most frequently injured joint in the human body, accounting for nearly 5% of emergency visits and about 40% of sports-related injuries [1,2]. Inversion or blunt trauma often results in ligament sprains, muscle strains, or fractures [2]. Acute lateral malleolar avulsion fractures (LMAFs) occur in about 15% of inversion sprains and, if missed, may lead to nonunion, chronic instability, and pain, necessitating stricter immobilization or surgery compared with simple sprains [3,4]. Small bone fragments near the lateral malleolus may also be subfibular ossicles (SFOs), either as accessory bones (os subfibulae) or as remnants of nonunion avulsion fractures [5,6]. SFO prevalence ranges from 0.2% to 6.7% and they are usually asymptomatic but appear in 10% to 38.5% of chronic ankle instability cases, suggesting a link to chronic dysfunction [7]. Both LMAF and SFO may cause lateral ankle pain and instability, making treatment selection more relevant than etiologic differentiation [8,9]. However, diagnosis is difficult because of overlapping clinical symptoms and ambiguous history or physical findings [10]. Radiography, the first-line imaging, has a missed fracture rate of 14% to 85% owing to small fragments, minimal displacement, suboptimal imaging angles, low quality, insufficient clinical context, and anatomical overlaps [11-15]. Although computed tomography (CT) improves visualization, large-scale validation for LMAF is lacking. While fragment morphology on CT can aid in distinguishing LMAF from SFO [16,17], this is complicated by smooth edges, incomplete separation, or osteophytes [18]. Magnetic resonance imaging (MRI) excels at detecting bone marrow edema and ligament injuries [19] but is limited by thick slices, partial volume effects, contraindications, and poor emergency availability. Thus, reliable and automated imaging-based diagnosis remains a key clinical need.

Deep learning and radiomics have achieved impressive results for the automated detection and classification of musculoskeletal fractures—including those of the ankle [20], femoral neck [21], hip [22], knee [23], spine [24], ribs [25], and scapula [26]—by using various deep convolutional neural network (DCNN) models, such as You Only Look Once (YOLO) and faster region-based convolutional neural network (R-CNN). Despite these advances, most existing studies focus on single-task or single-model frameworks, leaving performance uncertainties for tasks requiring differentiation of closely related lesions. To date, no DCNN-based approaches for automated detection of LMAF and SFO have been reported. With continuous architectural innovation, DCNNs are gaining traction for more complex orthopedic imaging tasks. Early studies focused on normal anatomy identification or simple diagnoses [27], but recent work extended to recognizing and classifying a variety of lesions—including multiple myeloma [28], patellar dislocation [29,30], osteoarthritis [31], bone age assessment [32], bone metastasis [33], and lumbar disk herniation [34]. However, automated detection and classification of LMAF and SFO in CT images remain challenging because of the relatively simple regional anatomy, diverse injury patterns, overlapping structures, and frequent imaging artifacts that even experienced radiologists find difficult. This challenge is more pronounced in primary hospitals lacking experienced musculoskeletal imaging specialists, where misdiagnosis rates are higher. Our study addresses this specific gap and aims to improve diagnostic accuracy and efficiency for this overlooked region using DCNNs.

### This Study

Studies using multi-model DCNN comparisons for detection and classification are still scarce. Comparative evaluation of mainstream architectures is valuable for understanding network performance and guiding the selection of optimal solutions for specific clinical tasks. YOLOv12, released by the Ultralytics team in February 2025, introduces substantial optimizations in network structure, feature fusion, and inference speed over its predecessors (eg, YOLOv8 and YOLOv9), achieving a better balance between accuracy and computational efficiency [35]. In this study, 4 advanced DCNN models—faster R-CNN, single shot multibox detector (SSD), RetinaNet, and the latest YOLOv12—were benchmarked for automated detection and classification of LMAF and SFO in multicenter clinical CT datasets. The models’ accuracy was quantitatively evaluated using metrics, such as mean average precision (mAP), and their diagnostic performance was compared with that of radiologists to assess clinical utility.

## Methods

### Ethical Considerations

The authors are accountable for all aspects of the work in ensuring that questions related to the accuracy or integrity of any part of the work are appropriately investigated and resolved. The study was conducted in accordance with the Declaration of Helsinki (as revised in 2013). This study was approved by the Institutional Ethics Committee of the Sir Run Run Hospital, Nanjing Medical University (2025-SRFA-626), and Air Force Medical Center, Air Force Medical University (2025-11-PJ01). The institutional ethics committees waived written informed consent in view of the retrospective nature of the study. All the procedures performed were part of routine care. All images were deidentified before use to protect patient privacy.

### Patient Selection

Our study used medical imaging data from 2 hospitals in China. For the development of the training and internal validation sets, CT and MRI data were collected from patients with ankle sprains treated at Air Force Medical Center, Air Force Medical University, between January 2014 and December 2024 (Textbox 1).

**Inclusion and exclusion criteria for the development of the training and internal validation sets.**

**Inclusion criteria**

- Age ≥18 years with a history of ankle sprain within the past 3 days
- CT images showing a single free bone fragment adjacent to the lateral malleolus (size ≥1 cm)
- Complete raw CT and MRI data, with both examinations performed within a 1-week interval
- Complete clinical information, including sex and age

**Exclusion criteria**

- Suspected infection- or tumor-related pathological fractures
- Hereditary skeletal disorders or skeletal dysplasia
- Previous ankle surgery
- Low image quality or artifacts

**Figure 1:**
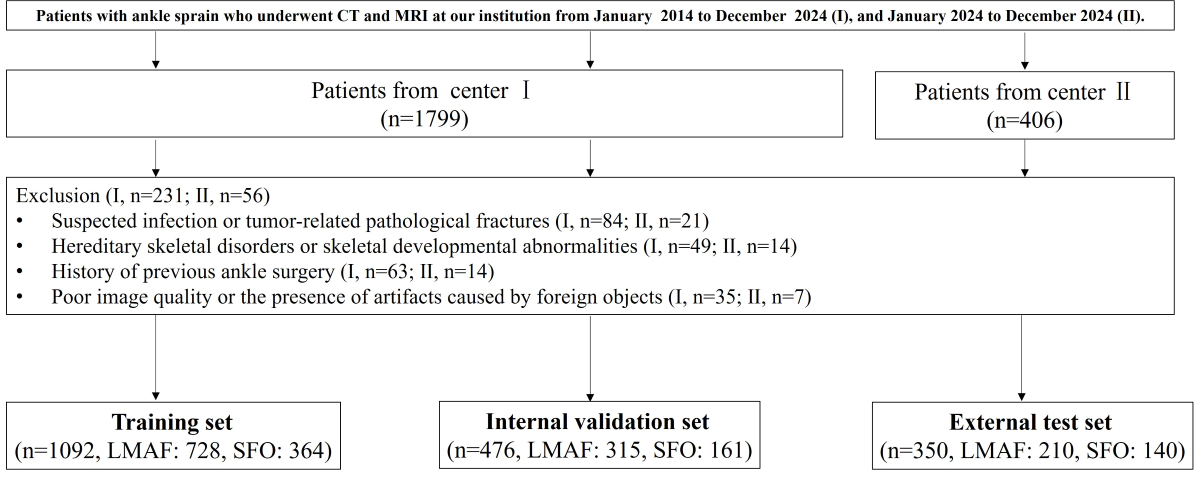
Flowchart summarizing patient selection and allocation to the dataset of our study.

An independent external test set was collected between January 2024 and December 2024 from Sir Run Run Hospital, Nanjing Medical University, using the same inclusion and exclusion criteria (Textbox 1). The detailed case selection process is illustrated in Figure 1. A total of 1918 patients were included in this study, comprising 1253 (65.3%) patients with LMAF and 665 (34.7%) with SFO. Of these, 1568 cases from the Air Force Medical Center were randomly allocated to a training set (n=1092, 56.93%) and an internal validation set (n=476, 24.82%) at a 7:3 ratio. The remaining 350 cases from Sir Run Run Hospital formed an independent external test set. Thus, the final training, internal validation, and external test sets contained 1092 (56.93%), 476 (24.82%), and 350 (18.25%) cases, respectively.

### CT and MRI Acquisition Protocol

Patient age and sex were obtained from the clinical record system. Details of CT and MRI acquisition devices and imaging parameters are provided in Multimedia Appendix 1. For CT scanning, patients were positioned supine with toes pointing upward and the ankle in a neutral position. The scan range included the distal tibia and fibula to the talus and surrounding bony structures (including the talus, calcaneus, and navicular). All images were reconstructed using a bone window (window width: 1500; window level: 500) with a slice thickness and interval of 1.0 mm. All image processing and analysis in our study were performed on the bone window of CT images.

### Classification

In this study, the initial differentiation between LMAF and SFO was made on the basis of MRI findings, which served as the reference standard for diagnosis. LMAF was defined as the presence of bone marrow edema in the corresponding region of the distal fibula on MRI, frequently accompanied by soft tissue swelling or evidence of ligamentous injury [36]. In contrast, SFO was characterized by the absence of bone marrow edema and lack of surrounding soft tissue injury, consistent with a chronic, stable ossicle. The interpretation of MRI images was independently performed by 2 senior radiologists, each with more than 20 years of experience in musculoskeletal imaging. The evaluation criteria included cortical continuity of the distal fibula, the margin and morphology of the bone fragment, and associated soft tissue changes. Any discrepancy between the 2 radiologists’ interpretations was resolved through consensus discussion. In cases where consensus could not be reached, a third experienced musculoskeletal radiologist was consulted, and the final diagnosis was established on the basis of a majority opinion.

### Evaluation of the Radiologists’ Visual Diagnoses Based on CT Images

For the external test set, each case was independently reviewed by radiologist 1 (a deputy chief radiologist with 15 years of musculoskeletal imaging experience) and radiologist 2 (an attending radiologist with 7 years of musculoskeletal imaging experience). Neither radiologist had access to the MRI results or clinical data during image interpretation. All images were evaluated independently by radiologists 1 and 2. Each radiologist recorded their classification of LMAF or SFO independently. For purposes of calculating diagnostic performance metrics, the diagnostic results of each radiologist were compared against the reference standard, which was established by MRI findings. Each radiologist’s performance on the external test set was then directly compared with that of the YOLOv12 model to evaluate whether the artificial intelligence (AI) model outperformed human assessment.

### Data Preparation

All CT images were acquired in Digital Imaging and Communications in Medicine (DICOM) format to ensure high resolution and rich grayscale information. Data preprocessing, a critical step in our study, included data selection, image format conversion, normalization, and data augmentation to meet the requirements for model development. Metadata, such as scanning parameters, slice thickness, and window width and level, were extracted using specialized tools to ensure data completeness. All images were converted to standardized PNG format while maintaining original resolution, facilitating subsequent size unification. To address grayscale distribution differences resulting from various scanners and settings, histogram equalization and pixel normalization were applied, mapping grayscale values to a fixed range. All images were resized to 640×640 pixels, providing consistent input dimensions for the models. To further increase data volume and enhance model robustness, data augmentation techniques such as random rotation, translation, scaling, and mirroring were used, generating additional training samples without compromising medical image integrity. This approach improved the model’s ability to recognize fracture features from various angles and positions. The entire preprocessing pipeline ensured uniform data quality and format, providing high-quality, well-annotated images for training, thereby improving the accuracy and robustness of the detection tasks.

**Figure 2:**
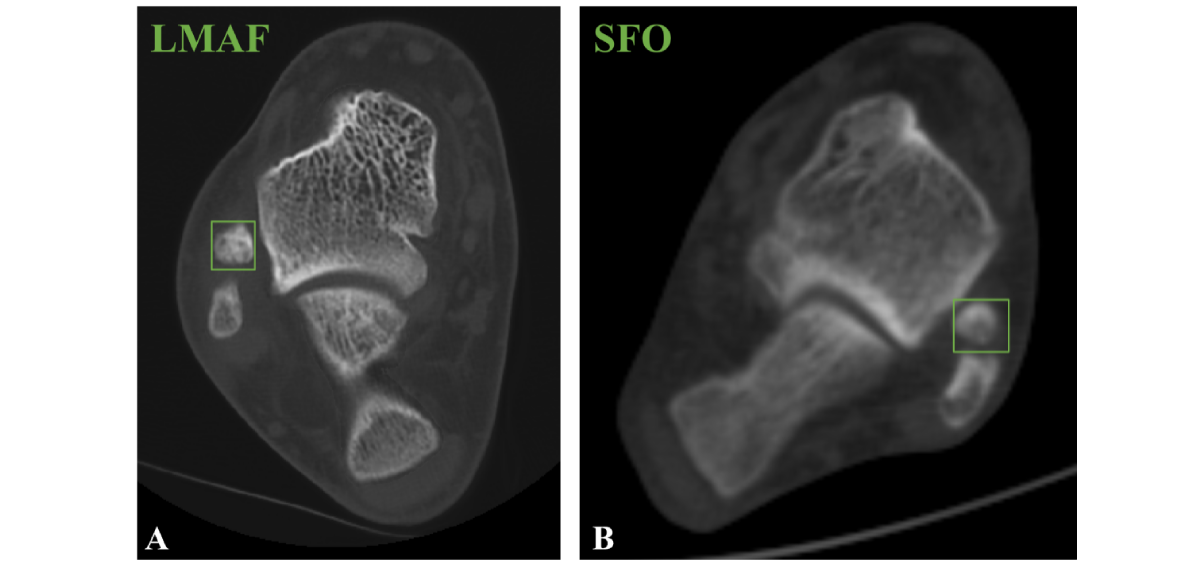
The lesions are labeled by drawing a tight bounding box. (A) An example of lateral malleolar avulsion fracture (LMAF). (B) An example of subfibular ossicle (SFO).

The annotation stage was performed independently by radiologists 1 and 2. Each lesion was labeled with a tight bounding box (Figure 2) using the Visual Geometry Group Image Annotator (VGG Image Annotator, University of Oxford). The fracture regions were manually outlined, and all annotations underwent cross-validation and quality control to ensure that each bounding box accurately represented the clinical lesion. To meet the input requirements of the detection models, we further processed the bounding boxes and cropped the images, ensuring that both the annotated regions and surrounding contextual information were consistently retained. This provided the models with sufficient and high-quality training samples.

### LMAF and SFO Detection Networks

**Figure 3:**
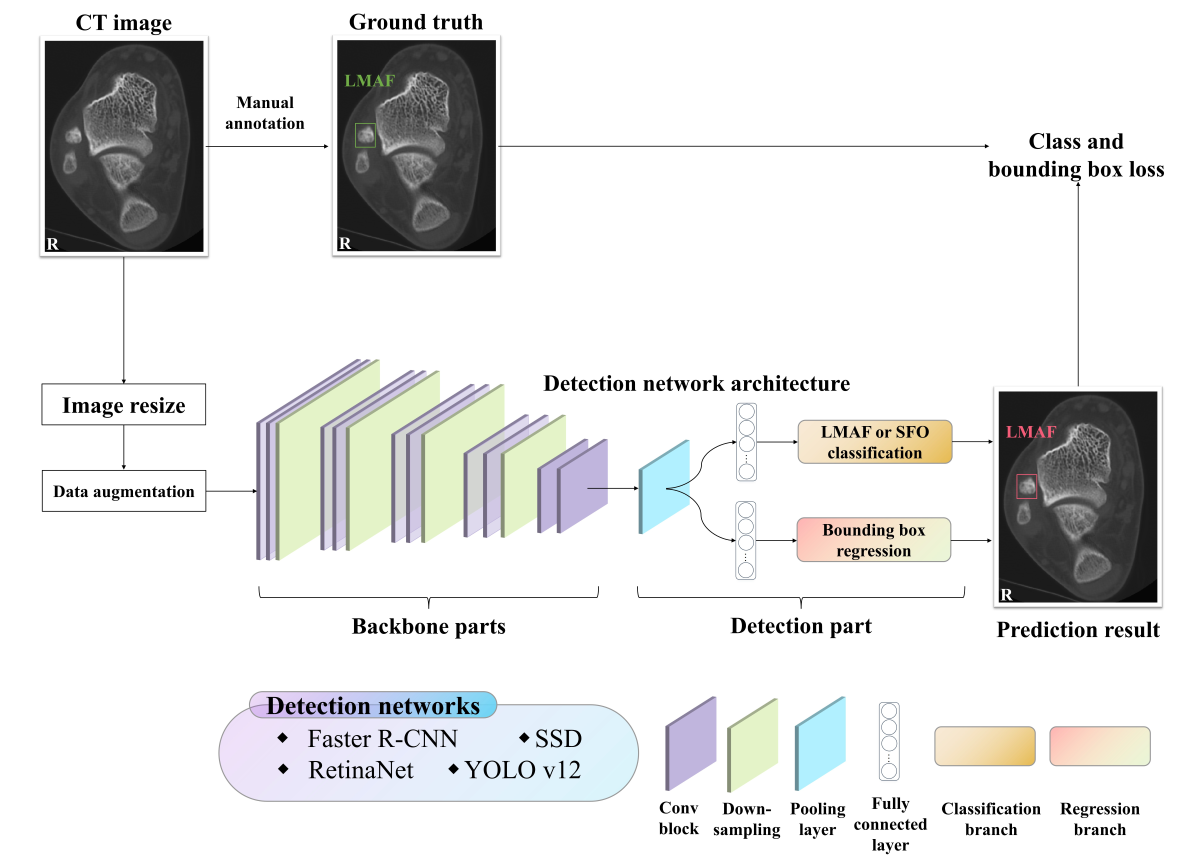
Schematic diagram of deep learning approaches for detection of lateral malleolar avulsion fracture (LMAF) and subfibular ossicle (SFO) on computed tomography (CT) images. R-CNN: region-based convolutional neural network; SSD: single shot multibox detector; YOLO: You Only Look Once.

In our study, 4 types of state-of-the-art object detection networks for LMAF and SFO detection on CT images were used (Figure 3).

**Figure 4:**
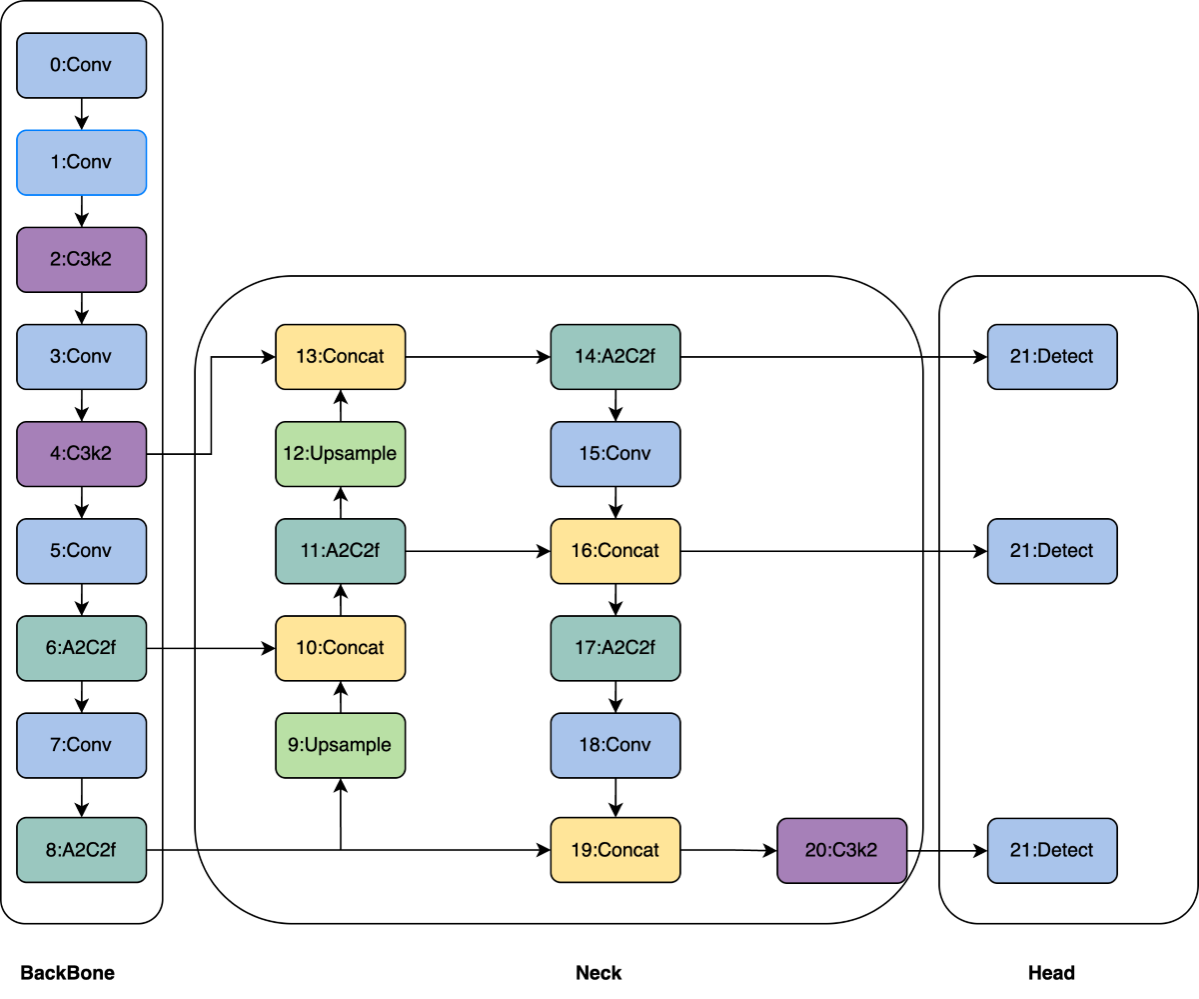
The model architecture of YOLOv12.

In this study, we used the YOLOv12 architecture, which was recently introduced on February 19, 2025, as the core detection network for LMAF and SFO identification (Figure 4). YOLOv12 advances previous one-stage detectors through a series of architectural innovations that balance high detection accuracy and efficient inference, which is critical in clinical and real-time applications. YOLOv12 is built upon a fully convolutional network backbone, eliminating fully connected layers to enhance both computational efficiency and spatial information retention. The network starts with a series of stacked convolutional and pooling layers in the front end, enabling progressive extraction of low-level features such as edges, textures, and local structures. This early feature extraction facilitates the precise localization of subtle abnormalities frequently present in medical images. Subsequently, multiple residual blocks capture higher-level semantic features, ensuring a comprehensive representation of features at different scales. YOLOv12 also incorporates a multi-scale feature fusion module, which integrates deep and shallow features via cross-layer connections, enabling precise localization in fine regions, such as fracture sites. In addition, an adaptive anchor box mechanism and multi-scale prediction strategy enable the model to achieve high performance in both classification and localization tasks. Finally, the introduction of a lightweight attention mechanism enhances the model’s focus on fracture edges and subtle structural features, improving detection accuracy and robustness while maintaining real-time inference capability.

To further boost localization reliability, YOLOv12 uses an adaptive anchor box mechanism. Anchor box sizes are dynamically learned from the training data distributions, optimizing the detection of objects with varying shapes and scales. In addition, a multi-scale prediction head enables simultaneous detection at 3 distinct spatial resolutions, allowing the model to maintain high precision when dealing with objects of different sizes. A notable architectural advancement is the integration of a lightweight attention mechanism based on coordinate attention modules. This enables the network to adaptively recalibrate feature responses, directing more focus toward critical areas—such as fracture boundaries or subtle morphological cues—while suppressing irrelevant background information. This not only improves detection performance, especially for challenging cases, but also preserves the real-time inference speed required in clinical workflows. All experiments were performed using the PyTorch 2.3 framework (Meta Platforms) on an NVIDIA RTX 4090 GPU (NVIDIA Corporation). The above hyperparameter choices were optimized by grid search to maximize the mAP on the validation subset. The main hyperparameter configurations were as follows: input image size: 640×640 pixels; batch size: 32; learning rate: initialized at 0.01 with cosine annealing schedule; optimizer: stochastic gradient descent with momentum 0.937 and weight decay 0.0005; anchor box generation: k-means clustering on the training data set; confidence threshold: 0.01; nonmaximum suppression intersection over union (IoU) threshold: 0.5; attention module: coordinate attention with a reduction ratio of 32; augmentation: mosaic, mix-up, and random affine transformations.

To comprehensively compare the performance of different models in the detection of LMAF and SFO, in addition to YOLOv12, we also implemented 3 other object detection models: faster R-CNN, SSD, and RetinaNet. Faster R-CNN, a representative 2-stage detection model, generates region proposals through a region proposal network, followed by refined classification and bounding box regression, providing a strong benchmark for localization accuracy. The SSD model uses a single-stage detection strategy, enabling high-speed detection while supporting multi-scale object detection. RetinaNet introduces the focal loss function to effectively address class imbalance and enhance the detection of small targets and subtle structures. All models were trained and tested using the same data preprocessing and augmentation strategies to ensure experimental comparability. In addition, specific hyperparameter tuning strategies were designed for each model.

### Training Setup

The detection networks were trained for 50 epochs with a batch size of 8. Data augmentation techniques applied during training included rotation (angle range −30° to 30°), width and height translation (horizontal and vertical axes: −20% to 20%), and scaling (range 0% to 20%). The networks were optimized using the AdamW optimizer with *β*_1_=0.9 and *β*_2_=0.999. The initial learning rate was set to 1×10⁻³ and reduced to 1×10⁻⁶ if the validation loss plateaued after 25 epochs. All deep learning models were implemented in Python 3 using the PyTorch framework (Meta Platforms) and trained on a workstation equipped with an Intel i9–7900X CPU (Intel Corporation; 3.3 GHz), 128 GB RAM, and 2 NVIDIA TITAN RTX GPU (NVIDIA Corporation). All models were trained and tested under identical computational conditions, using the same data augmentation pipeline and 3 consistent hyperparameters, ensuring an unbiased comparison across models.

### Evaluation Metrics and Vision Transformer Shapley Saliency Maps

An end-to-end training architecture was adopted throughout the network design, with joint optimization of module parameters achieved via error backpropagation. To evaluate the detection performance for LMAF and SFO, we used the following metrics: mAP, average precision (AP), precision, recall, and *F*_1_- score. AP was determined by the area under the precision-recall (PR) curve, which was constructed based on the maximum overlap (IoU) between detected and ground-truth bounding boxes. The PR curve is a key tool for assessing the performance of classifiers or object detection models, especially in the context of class imbalance, by visualizing the trade-off between precision and recall across different decision thresholds. Detection time was also measured for a comprehensive performance comparison, aiming to provide multidimensional insights for the clinical application of automated LMAF and SFO detection.

The mAP measures the model’s AP across different classes and IoU thresholds, and we calculated the AP at an IoU threshold of 0.5 (ie, mAP50). Please refer to Multimedia Appendix 2 for the calculation method. To further interpret the model’s decision process, saliency maps were generated using Shapley techniques [37]. Receiver operating characteristic curves and the AUC were plotted and calculated to assess model performance, and the DeLong test was used to compare AUCs between models. Calibration curves and decision curve analysis were computed and plotted using the *RMS* and *rmda* packages in R software (version 4.0.2), and the Hosmer-Lemeshow test was used to evaluate calibration. For radiologists 1 and 2, diagnostic performance metrics—including sensitivity, specificity, accuracy, and AUC—were calculated for LMAF and SFO diagnosis, and the DeLong test was used to determine whether differences in AUC were significant. A *P* value <.05 was considered statistically significant. The statistical analyses were carried out using our internally developed PixelMedAI platform. This platform was programmed in Python version 3.8 and primarily used libraries, such as PyTorch, NumPy, and SciPy. This approach ensures a robust evaluation of both AI models and human experts, facilitating a comprehensive understanding of the effectiveness of saliency maps in enhancing diagnostic accuracy in clinical settings.

## Results

### Clinical Features of the Studied Patients

A total of 1918 patients met the inclusion criteria, comprising 1253 (65.3%) cases of LMAF and 665 (34.7%) cases of SFO. The patients ranged in age from 18 to 70 years, with a mean age of 33.52 (SD 12.83) years. There were 1463 male patients (76.3%) and 355 female patients (23.7%). The training set, internal validation set, and external test set included 1092 (56.93%) cases (LMAF: 728, SFO: 364), 476 (24.82%) cases (LMAF: 315, SFO: 161), and 350 (18.25%) cases (LMAF: 210, SFO: 140), respectively. The demographic characteristics of each data set are summarized in Table 1.

**Table 1:** Baseline characteristics of patients in the training set, internal validation set and external test set.

Characteristics			Training set (n=1092)		Internal validation set (n=476)		External test set (n=350)
Age (years), mean (SD)			31.47 (11.57)		32.19 (11.68)		34.94 (13.03)
**Sex, n (%)**							
	Male	847 (77.6)		364 (76.4)		252 (72)	
	Female	245 (22.4)		112 (23.6)		98 (28)	

### Performance of DCNNs

**Figure 5:**
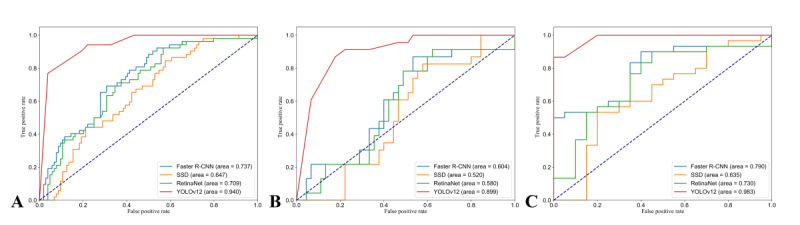
Precision and recall curves of the 4 deep convolutional neural networks are displayed in different colors, with the performance of each model shown. YOLOv12 achieved the highest performance, as demonstrated in its precision and recall curve. (A) Training set. (B) Internal validation set. (C) External test set. R-CNN: region-based convolutional neural network; SSD: single shot multibox detector; YOLO: You Only Look Once.

A comparative analysis of the detection performance of the 4 DCNNs models was conducted. Figure 5 and Multimedia Appendix 3 show the PR curves and receiver operating characteristic (ROC) curves for each model, respectively. The mAP values were compared to evaluate the performance of each DCNN in detecting LMAF and SFO on CT images. In the test set, the mAP50 scores for faster R-CNN, SSD, RetinaNet, and YOLOv12 were 63.7%, 63.0%, 67.0%, and 92.1%, respectively. The AUC values for these models were 0.79 (faster R-CNN), 0.63 (SSD), 0.73 (RetinaNet), and 0.98 (YOLOv12). Pairwise comparisons using the DeLong test indicated that the differences were all statistically significant (*P*<.001). These results demonstrate that YOLOv12 achieved the highest performance in LMAF and SFO detection. The calibration curves for the detection performance of the 4 DCNNs on CT images are shown in Multimedia Appendix 4. The calibration curves demonstrated that the predicted probabilities closely matched the actual probabilities for the training, internal validation, and external test sets (Hosmer-Lemeshow test: *χ*^2^=8.2, *χ*^2^=2.6, *χ*^2^=22.7, respectively; *P*=.21, *P*=.46, *P*=.13, respectively). Decision curve analysis results indicate that YOLOv12 may serve as an effective tool for detecting LMAF and SFO (Multimedia Appendix 5).

### Performance Between the YOLOv12 Model and Radiologists’ Readings

Table 2 shows the AUC, sensitivity, specificity, and accuracy of YOLOv12 and radiologists 1 and 2 in detecting LMAF and SFO. The AUCs for YOLOv12, radiologist 1, and radiologist 2 were 0.983, 0.755, and 0.682, respectively. Pairwise comparisons using the DeLong test revealed statistically significant between radiologist 1, radiologist 2, and YOLOv12 (*P*=.004, *P*<.001), indicating that YOLOv12 outperformed both radiologists.

**Table 2:** Comparison of AUC, accuracy, sensitivity, and specificity between the YOLOv12 model and radiologists’ readings.

	AUC	Accuracy	Sensitivity	Specificity	*P* value
YOLOv12	0.983	0.920	0.867	0.822	–
Radiologist 1	0.755	0.756	0.750	0.760	.004
Radiologist 2	0.682	0.691	0.652	0.711	<.001

### Evaluation of Saliency Maps

**Figure 6:**
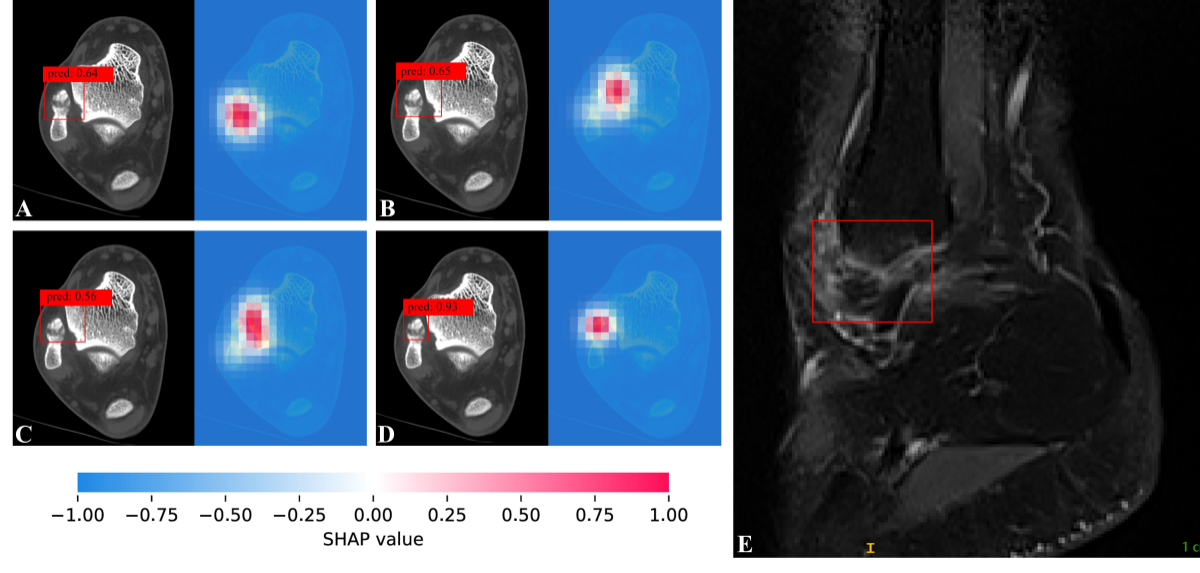
Case of lateral malleolar avulsion fracture. By using the ViT Shapley technique, we generated a saliency map where the red highlighted areas roughly correspond to the lateral malleolar avulsion fracture seen in the magnetic resonance imaging image. (A) Faster region-based convolutional neural network. (B) Single shot multibox detector. (C) RetinaNet. (D) YOLOv12. (E) Sagittal PDWI-weighted magnetic resonance imaging image of the ankle.

**Figure 7:**
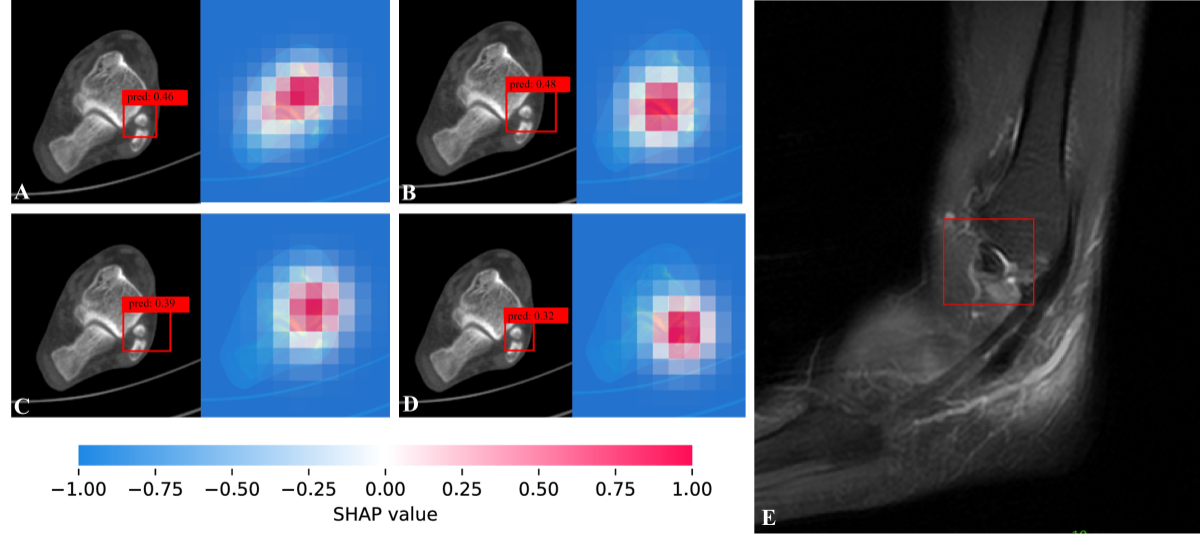
Case of subfibular ossicle. By using the ViT Shapley technique, we generated a saliency map where the red highlighted areas roughly correspond to the subfibular ossicle seen in the magnetic resonance imaging image. (A) Faster region-based convolutional neural network. (B) Single shot multibox detector. (C) RetinaNet. (D) YOLOv12. (E) Sagittal PDWI-weighted magnetic resonance imaging image of the ankle.

Saliency maps generated using the Shapley technique were used to highlight the region’s most influential for predicting LMAF (Figure 6) and SFO (Figure 7), with red pixels indicating areas with the greatest impact on the model’s predictions. As shown in Figure 6D, YOLOv12 assigned a relatively high probability (0.93) to LMAF, and the saliency map correspondingly highlights the LMAF region. In Figure 7D, YOLOv12 assigned a relatively low probability (0.32) to LMAF, indicating a higher probability for SFO, and the saliency map highlights the SFO region accordingly.

## Discussion

### Principal Findings

Our study is the first to systematically evaluate and compare multiple mainstream DCNNs for the automatic detection and classification of LMAF and SFO on ankle CT images. Among the models tested, YOLOv12 demonstrated the highest detection accuracy and efficiency, with mAP50 of 92.1% and AUC of 0.98 on the external validation cohort. When compared with radiologists 1 and 2 with different experience levels, YOLOv12 surpassed human performance in accuracy, sensitivity, and specificity, highlighting the powerful potential of deep learning for recognizing subtle and complex anatomical structures. Quantitative analysis further confirmed the model’s high reproducibility and stability, effectively addressing limitations of human interpretation, such as inconsistency and time consumption. Most errors were associated with confusing anatomical variations and inaccurate localization of lesion boundaries, but overall, the model met the practical needs for clinical auxiliary diagnosis and provided a solid technical foundation for intelligent diagnosis in orthopedics. In keeping with real-world clinical workflows—where LMAF and SFO may coexist and readers are expected to detect all relevant abnormalities within a single study—our primary evaluation focused on performance in the combined data set.

### Comparison to Prior Work

Prior work using object detection networks—such as faster R-CNN, RetinaNet, or earlier YOLO versions (eg, YOLOv3 to YOLOv8)—has achieved promising results for fracture localization in radiographs, but performance often declined in cases involving small, subtle lesions or overlapping anatomical structures [26,38,39]. Furthermore, these studies were typically limited by homogeneous datasets, single-center validation, or a lack of rigorous comparison to clinical radiologist interpretation. Our study addresses these gaps by introducing a comprehensive, multicenter evaluation of several state-of-the-art DCNN architectures, including the newly released YOLOv12 model. Notably, YOLOv12 incorporates advanced multiscale feature fusion and optimized attention mechanisms, resulting in substantial improvements in both detection accuracy and computational efficiency over previous frameworks [40]. Compared with earlier works, our head-to-head benchmarking on large-scale, well-annotated multicenter CT data sets demonstrates that YOLOv12 outperformed established models, such as faster R-CNN, SSD, and RetinaNet in terms of mAP, AUC, sensitivity, and specificity. Moreover, unlike most previous research—which reported model performance in isolation—our study directly compares the diagnostic capability of deep learning models with radiologists of differing clinical experience. YOLOv12 demonstrated superior accuracy (92% vs 75.6% and 69.1%) and AUC (0.98 vs 0.76 and 0.68) over 2 musculoskeletal imaging specialists, underscoring its clinical potential for assisting or augmenting human diagnosis, particularly in settings where radiological expertise may be limited.

### Performance Interpretation

In this study, the YOLOv12-based object detection model achieved a mAP of 92.1% at an IoU threshold of 0.5 (mAP50) on the test set, indicating that the model was able to accurately detect 92.1% of actual lesion regions when the IoU threshold was set to 0.5. Specifically, this metric reflects the proportion of predicted bounding boxes whose overlap with the ground-truth annotated lesion regions reached or exceeded 50%. Although this result demonstrates the model’s strong performance in localizing lesion targets, some differences remained between the predicted and actual regions in terms of size and shape, mainly because of the relatively lenient IoU threshold. Such discrepancies are common in practical applications; nevertheless, the model is largely capable of accurately capturing key lesion information to meet the requirements of subsequent diagnostic assistance. The automatic detection and classification capabilities of YOLOv12 exhibited high reproducibility and stability, effectively compensating for the limitations of manual interpretation in terms of repeatability and efficiency, thereby providing more reliable technical support for clinical diagnosis. YOLOv12 incorporates advanced attention mechanisms and a multi-branch feature fusion structure, enabling it to efficiently capture object information at different scales and under complex scenarios [41]. Furthermore, its optimized network architecture and lightweight design significantly reduced the number of model parameters and computational load while maintaining high detection performance, balancing inference speed with resource use efficiency. This makes it suitable for edge deployment and large-scale real-time detection tasks in actual applications.

### Detection Errors and Challenges

In our study, the first main error observed with the YOLOv12 model was the misclassification of SFO as LMAF, resulting in false positives. This misjudgment was primarily caused by confusion with normal anatomical structures or adjacent soft tissue shadows, as some anatomical variants or features of the fibula could easily be mistaken for pathological findings by the network [42]. These misleading regions also pose a considerable challenge for human readers, reflecting the DCNN’s sensitivity to areas with “clear boundaries and interrupted bone continuity” during the learning process [43], which closely mirrors the clinical approach used by physicians. Therefore, many of the model’s false positives overlapped with the subtle or ambiguous cases that even experienced clinicians might misinterpret.

The second error type involved correct detection of the presence of abnormal structures, but imprecise localization of the region of interest. In these instances, the model could identify that a loose fragment or abnormal bone structure existed, yet failed to delineate the precise borders or classify the fragment correctly, particularly regarding its exact size, avulsion site, or associated ligament anatomy. Further analysis revealed that while DCNNs are generally adept at capturing the spatial relationship between fractures, joints, and adjacent bone structures, they remain less effective in distinguishing finer anatomical details [44]. Cases with subtle displacement, overlapping structures, or minimal contrast differences contributed to these localization issues. Future improvements could be achieved by integrating detection networks with training datasets that include enhanced annotation of normal anatomical variations, thereby reducing both false-positive and false-negative rates through a more nuanced understanding of the relevant anatomical context [45].

### Limitations and Future Directions

Our study still has some limitations. First, when comparing the performance of the YOLOv12 with that of radiologists for automatic detection and classification of LMAF and SFO on CT images, the experimental design required informing the radiologists in advance about the specific lesion types and regions of interest. This likely improved the diagnostic accuracy of the radiologists and resulted in an optimistically biased representation of manual interpretation. In real clinical practice, some lesions are easily missed or misdiagnosed, so this “prompt” provided a considerable advantage to the physicians. Second, this study did not include complex cases with multiple coexisting lesions, which reduced the amount of data available for model training. Future studies should incorporate such complex or multifocal cases to better evaluate the accuracy of DCNNs in clinical scenarios that more closely resemble real-world situations. Third, all imaging data in this study were collected from only 2 medical institutions. Although multiple scanners and extensive data augmentation were used to minimize bias, this may still affect the generalizability of the model to other institutions and populations. To further improve the model’s applicability and robustness, future work should involve validation with multicenter, multi-scanner, and multisource datasets [46]. Fourth, while our design enhances clinical realism, it limits the granularity of class-specific assessment; we did not perform separate training or testing on LMAF-only and SFO-only datasets or report per-class mAP. As our dataset expands, we plan to conduct category-specific experiments and provide detailed per-class detection metrics to further delineate class-dependent performance and error modes.

### Conclusions

Our study developed a DCNN-based method for automatic detection and classification of LMAF and SFO in CT images. The results showed that the YOLOv12 network outperformed mainstream models such as faster R-CNN, SSD, and RetinaNet in both detection and classification tasks. In addition, the YOLOv12 model achieved higher classification accuracy than radiologists.

**Acknowledgments**

This work was supported by Medical Scientific Research Project of the Jiangsu Provincial Health Commission (grant M2024055) and the Medical Imaging Artificial Intelligence Special Research Fund Project, Nanjing Medical Association Radiology Branch (grant 16). We express our gratitude to the editor and anonymous reviewers for their insightful comments and suggestions, which have greatly enhanced the quality of our paper. We also extend our thanks to American Journal Experts for their assistance in editing the language of an earlier draft of this manuscript. We appreciate the guidance and support from the PixelMedAI platform and its developers concerning the code used in this revised manuscript.

## Appendix

Multimedia Appendix 1 The computed tomography (CT) and magnetic resonance imaging (MRI) image acquisition parameters of the 2 centers.

Multimedia Appendix 2 Supplementary formula.

Multimedia Appendix 3 Receiver operating characteristic curves of 4 deep convolutional neural network (DCNN) models (A: training set; B: internal validation set; C: external test set).

Multimedia Appendix 4 Calibration curve for the prediction model (A: training set; B: internal validation set; C: external test set).

Multimedia Appendix 5 Decision curve analysis of the model (A: training set; B: internal validation set; C: external test set).

## Abbreviations

AI: artificial intelligence
AP: average precision
AUC: area under the curve
CT: computed tomography
DCNN: deep convolutional neural network
DICOM: Digital Imaging and Communications in Medicine
IoU: intersection over union
LMAF: lateral malleolar avulsion fracture
mAP: mean average precision
mAP50: mean average precision at intersection over union 0.5
MRI: magnetic resonance imaging
PR: precision-recall
R-CNN: region-based convolutional neural network
ROC: receiver operating characteristic
SFO: subfibular ossicle
SSD: single shot multibox detector
YOLO: You Only Look Once

## Data Availability

The datasets generated or analyzed during this study are not publicly available because of privacy or ethical restrictions but are available from the corresponding author upon reasonable request.
