# Supplementary material for: YOLOv12 Algorithm-Aided Detection and Classification of Lateral Malleolar Avulsion Fracture and Subfibular Ossicle Based on CT Images: Multicenter Study
Source: JMIR Med Inform. 2025 Oct 3;13:e79064. doi: 10.2196/79064 (PMC12534769; doi:10.2196/79064)
Supplement: Multimedia Appendix 1 [file medinform_v13i1e79064_app1.docx]

|  |  | Center I | Center Ⅱ |
| --- | --- | --- | --- |
|  | Parameters | Air Force Medical Center,  Air Force Medical University | Sir RunRun Hospital,  Nanjing Medical University |
| CT system information | CT system | 16-slice multi-detector CT scanner (Lightspeed Ultra, GE Healthcare, USA) or 128-slice Dual source CT (Somatom Definition, Siemens Healthcare, Germany) or 64-slice spiral CT (SOMATON sensation64, Siemens Healthcare, Germany) | 256-slice spiral CT (Brilliance iCT, ROYAL PHILIPS, Netherlands) or 64- slice multi-detector CT (Optima CT670, GE Healthcare, USA) |
| CT scan parameters | Tube voltage | 120 kVp with  automated tube current modulation | 120 kVp with  automated tube current modulation |
|  | Tube current | 125 – 310 mA or 185 mA with automated tube current modulation | 180 mA with automated tube current modulation |
| CT image information | Image matrix | 512×512 | 512×512 |
|  | layer thickness and layer interval | 1 mm, 1 mm | 1 mm, 1 mm |
| MRI system information | MRI system | Verio 3.0T (Siemens Healthcare, Germany) or Skyra 3.0T (Siemens Healthcare, Germany) or Ingenia CX 3.0T (PHILIPS, Netherlands) | Ingenia CX 3.0T (PHILIPS, Netherlands) or Magnetom Verio 3.0T (Siemens Healthcare, Germany) |
| MRI scan parameters | Axial PDWI sequence | TR/TE of 2772 ms/38 ms,  slice thickness of 3 mm,  interslice gap of 10 mm,  FOV of 160 × 160 mm. | TR/TE of 3312 ms/30 ms,  slice thickness of 3 mm,  interslice gap of 10 mm,  FOV of 120 × 120 mm. |
|  | Sagittal PDWI sequence | TR/TE of 2432 ms/38 ms,  slice thickness of 3 mm,  interslice gap of 10 mm,  FOV of 160 × 160 mm. | TR/TE of 2649 ms/30 ms,  slice thickness of 3 mm,  interslice gap of 10 mm,  FOV of 140 × 140 mm. |
|  | Coronal PDWI sequence | TR/TE of 2432 ms/38 ms,  slice thickness of 3 mm,  interslice gap of 10 mm,  FOV of 160 × 160 mm. | TR/TE of 2129 ms/30 ms,  slice thickness of 3 mm,  interslice gap of 10 mm,  FOV of 160 × 160 mm. |
|  | Sagittal T1WI sequence | TR/TE of 500 ms/7.6 ms,  slice thickness of 3 mm,  interslice gap of 10 mm,  FOV of 160 × 160 mm. | TR/TE of 479 ms/8.3 ms,  slice thickness of 3 mm,  interslice gap of 10 mm,  FOV of 140 × 140 mm. |
