# Supplementary material for: YOLOv12 Algorithm-Aided Detection and Classification of Lateral Malleolar Avulsion Fracture and Subfibular Ossicle Based on CT Images: Multicenter Study
Source: JMIR Med Inform. 2025 Oct 3;13:e79064. doi: 10.2196/79064 (PMC12534769; doi:10.2196/79064)
Supplement: Multimedia Appendix 2 [file medinform_v13i1e79064_app2.docx]

$$Precision=\frac{TP}{TP+FP}\times100\%$$

TP: True Positives, FP: False Positives

$$Recall=\frac{TP}{TP+FN}\times100\%$$

FN: False Negatives

$$AP=\sum_{rk} r\times P(rk)$$

P(rk): The precision value at recall rate rk, △r: The difference between recall rates.

$$mAP50=\frac{1}{N}\sum_{i=1}^{N} AP_{i}$$

N is the total number of classes, and AP_i_ is the average precision for class i.

$$F1=2\cdot\frac{Precision\times Recall}{Precision+Recall}$$
