# Supplementary figures and images for: YOLOv12 Algorithm-Aided Detection and Classification of Lateral Malleolar Avulsion Fracture and Subfibular Ossicle Based on CT Images: Multicenter Study
Source: JMIR Med Inform. 2025 Oct 3;13:e79064. doi: 10.2196/79064 (PMC12534769; doi:10.2196/79064)

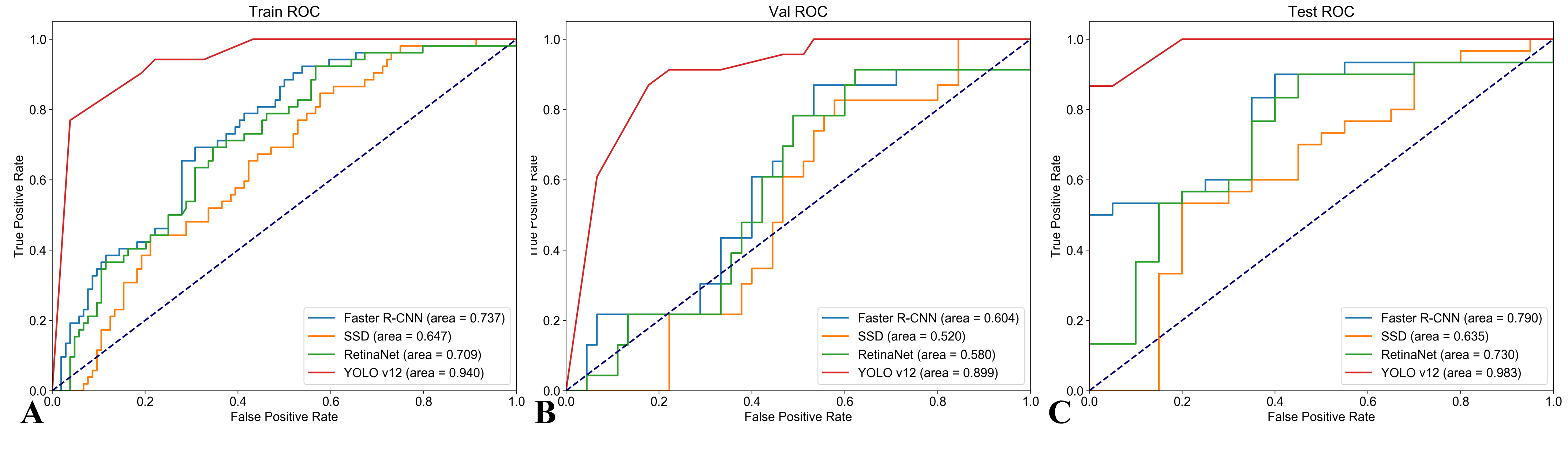

Supplement: Multimedia Appendix 3 [file medinform_v13i1e79064_app3.png]

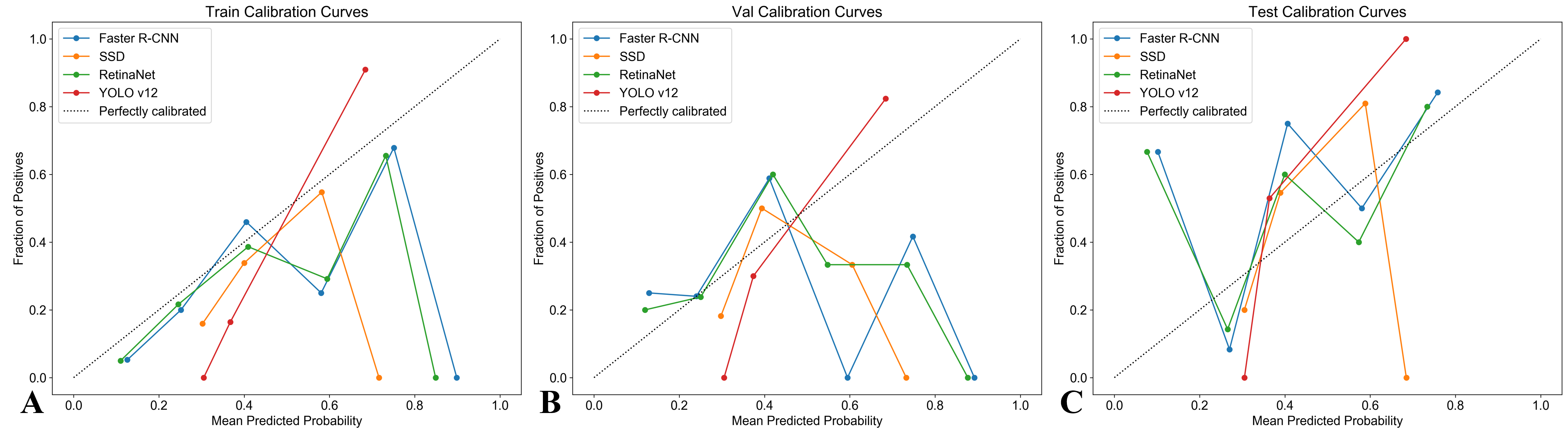

Supplement: Multimedia Appendix 4 [file medinform_v13i1e79064_app4.png]

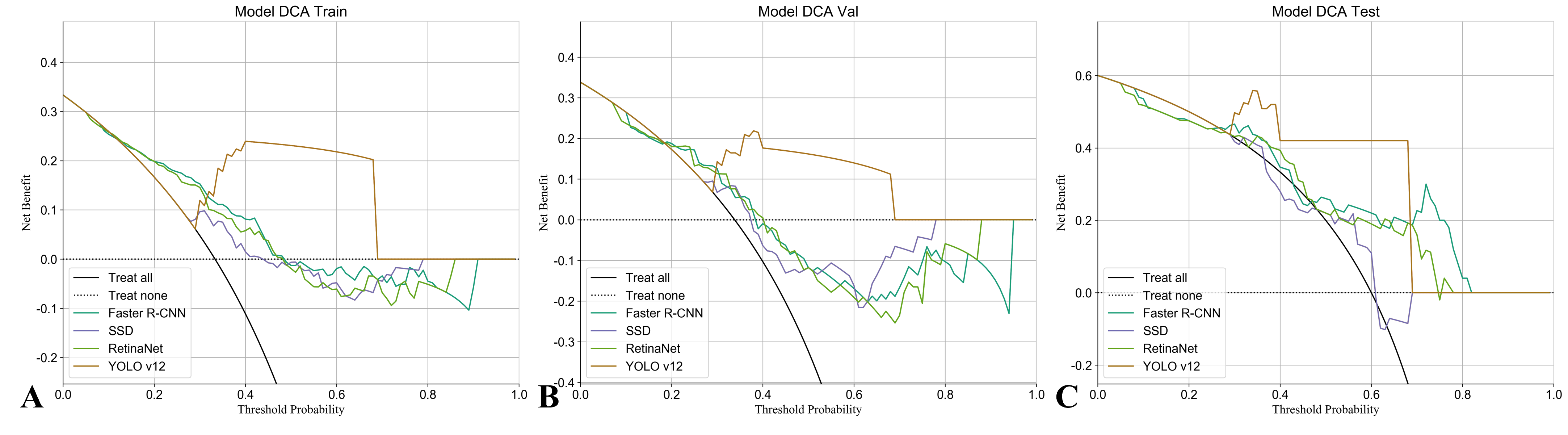

Supplement: Multimedia Appendix 5 [file medinform_v13i1e79064_app5.png]
